# Continuous Dissolved Oxygen Measurements and Modelling Metabolism in Peatland Streams

**DOI:** 10.1371/journal.pone.0161363

**Published:** 2016-08-24

**Authors:** Jonathan J. Dick, Chris Soulsby, Christian Birkel, Iain Malcolm, Doerthe Tetzlaff

**Affiliations:** 1 Northern Rivers Institute, School of Geosciences, University of Aberdeen, Aberdeen, AB24 3UF, United Kingdom; 2 University of Costa Rica, Department of Geography, 2060 San José, Costa Rica; 3 Marine Science Scotland, Freshwater Laboratory, Pitlochry, Scotland, United Kingdom; Louisiana State University, UNITED STATES

## Abstract

Stream water dissolved oxygen was monitored in a 3.2km^2^ moorland headwater catchment in the Scottish Highlands. The stream consists of three 1^st^ order headwaters and a 2^nd^ order main stem. The stream network is fringed by peat soils with no riparian trees, though dwarf shrubs provide shading in the lower catchment. Dissolved oxygen (DO) is regulated by the balance between atmospheric re-aeration and the metabolic processes of photosynthesis and respiration. DO was continuously measured for >1 year and the data used to calibrate a mass balance model, to estimate primary production, respiration and re-aeration for a 1^st^ order site and in the 2^nd^ order main stem. Results showed that the stream was always heterotrophic at both sites. Sites were most heterotrophic in the summer reflecting higher levels of stream metabolism. The 1^st^ order stream appeared more heterotrophic which was consistent with the evident greater biomass of macrophytes in the 2^nd^ order stream, with resulting higher primary productivity. Comparison between respiration, primary production, re-aeration and potential physical controls revealed only weak relationships. However, the most basic model parameters (e.g. the parameter linking light and photosynthesis) controlling ecosystem processes resulted in significant differences between the sites which seem related to the stream channel geometry.

## Introduction

Peatlands cover 3 million km^2^ of the Earth’s land surface and are characterised by their high rates of organic matter accumulation creating a water-retentive landscape [1]. Stream channel networks usually have a high drainage density and are highly connected to the wet soils, while stream channel morphology is closely linked to the ecohydrology of peatland structure [2]. Such streams act as a transport pathway of organic matter fluxes from the landscape and to downstream waters [3]. This organic matter is an important energy source for aquatic micro-organisms, and as such, its dynamism may influence metabolic processes [4,5]. In the UK, peatlands cover 12% of the land surface, they are common in upland areas [6] and are often located in the headwaters of many major river systems as saturated riparian areas fringing stream channels [7]. Metabolic processes in such headwater streams are poorly understood and need to be better characterised, particularly to aid sustainable land and water management [8–10]. The underlying processes of primary production and respiration are fundamental controls on the structure and function of lotic ecosystems [11]. These processes reflect the synthesis and breakdown of organic matter and as such are closely coupled to the biogeochemical cycles of both macro- and micronutrients that connect the landscape and freshwater environment [4];[5]. They also indicate the trophic state of the system [12,13] as indicated by photosynthesis/respiration ratio which is >1 or <1 for autotrophic or heterotrophic systems respectively [13]. Heterotrophic systems require an external supply of organic matter [14] and can become net sources of carbon to the environment. Most northern latitude streams are heterotrophic reflecting the large organic matter inputs and carbon storage in the organic rich peats present [15].

Recent developments in sensing technology have made long-term, accurate measurements of dissolved oxygen (DO) dynamics in aquatic systems both relatively easy and inexpensive [16]. Using such continuous time series of in-stream DO, stream metabolism can be estimated using open-channel methods [17]. These include a two-station method, which measures the downstream change in DO between two points [18]. A second approach is the one-station method, which uses the change in DO over a fixed time period at the same point [19]. The calculation of re-aeration is a key requirement and one of the greatest challenges in the use of open channel methods to estimate stream metabolism [20,21], and often the largest source of uncertainty [22]. Several methods for estimation are in common use, for example: propane injection for direct measurement [23], the night time regression method [24], and empirical models as reviewed by Aristegi et al. [22]. More recently, re-aeration has been derived either as a calibrated parameter in oxygen mass balance models (e.g. [25,26]), and can be used across a wide range of conditions [27] or using tracers to quantify the re-aeration coefficient (e.g. [28,29]). An essential need in the use of a calibrated parameter set is to control model uncertainty. Holtgrieve et al. [25] utilizes a formal uncertainty analysis, and Birkel et al. [26] constrains the calibration with model rejectionist framework to accept models with only a high likelihood of realism.

Here, we apply the methods of Birkel et al. [26] which iteratively calibrates re-aeration to individual 24hr periods using oxygen deficits. The calibrated re-aeration is then used to estimate metabolism. The focus will be on 1^st^ and 2^nd^ order headwater streams draining an upland peatland which contribute critical ecosystem services to a major river system downstream [30]. In the UK, peat streams usually drain un-forested moorlands and are typically characterised by open channels and dwarf shrubs and grasses as riparian vegetation, which gives limited riparian shading [31]. Riparian cover can have a major influence on metabolism by regulating incoming radiation [8]. The study is based in the 3.2km^2^ Bruntland Burn catchment which drains into the 31km^2^ Girnock catchment. Here a fully calibrated model was used to estimate stream metabolism in a comparison of forested and moorland reaches of a larger 3^rd^ order stream [26]. In this paper, we advance our research with a smaller scale process study, using the modelling approach of Birkel et al. [26] to infer stream metabolism in low order peatland streams. The specific hypotheses that we will test are:

1. That a calibrated mass balance model can simulate DO concentrations and metabolism in peatland streams.
2. That model successes and failures will provide insights into the relative importance of processes affecting metabolism in peat streams.

### Study Site

The Bruntland Burn is located in the Cairngorms National Park, Scotland, UK and has been described in detail elsewhere (e.g. [32,33]). The stream network comprises 1^st^ and 2^nd^ order channels (Table 1), which are typically narrow (0.5–1.5m) and relatively deep (0.5–2m), and for most of its length is bounded by peat soil. Stream chemistry at the site is circum-neutral (pH 6.0–7.5) and is nutrient poor; N and P levels are usually below detection, but flushes of N occur in the autumn [34]. The peat soils result in high dissolved organic carbon (DOC) concentrations (>10 mg l^-1^) which discolours the water during higher flows, most notably in summer [35]. Within the channel network we selected two sites to measure DO and estimate metabolism. Table 1 shows the catchment sizes, mean dissolved oxygen and water temperature for each of the study locations, which are situated in the upper catchment (UC1-2) on a first order stream and in lower catchment (LC1-2) on a second order channel.

**Table 1:** Catchment size for each optode and average dissolved oxygen concentration and stream temperature for the common period of study.

Optode	Catchment area (km^2^)	Average DO and *Standard Deviation* (mg l^-1^)	Average stream temperature and *Standard Deviation* (°C)	Channel width (m)
UC1	0.65	9.49 *(1*.*47)*	5.96 *(5*.*26)*	1.55
UC2		9.93 *(1*.*47)*		1.10
LC1	2.80	11.07 *(1*.*59)*	5.79 *(4*.*71)*	1.50
LC2		11.09 *(1*.*54)*		0.60

**Fig 1:**
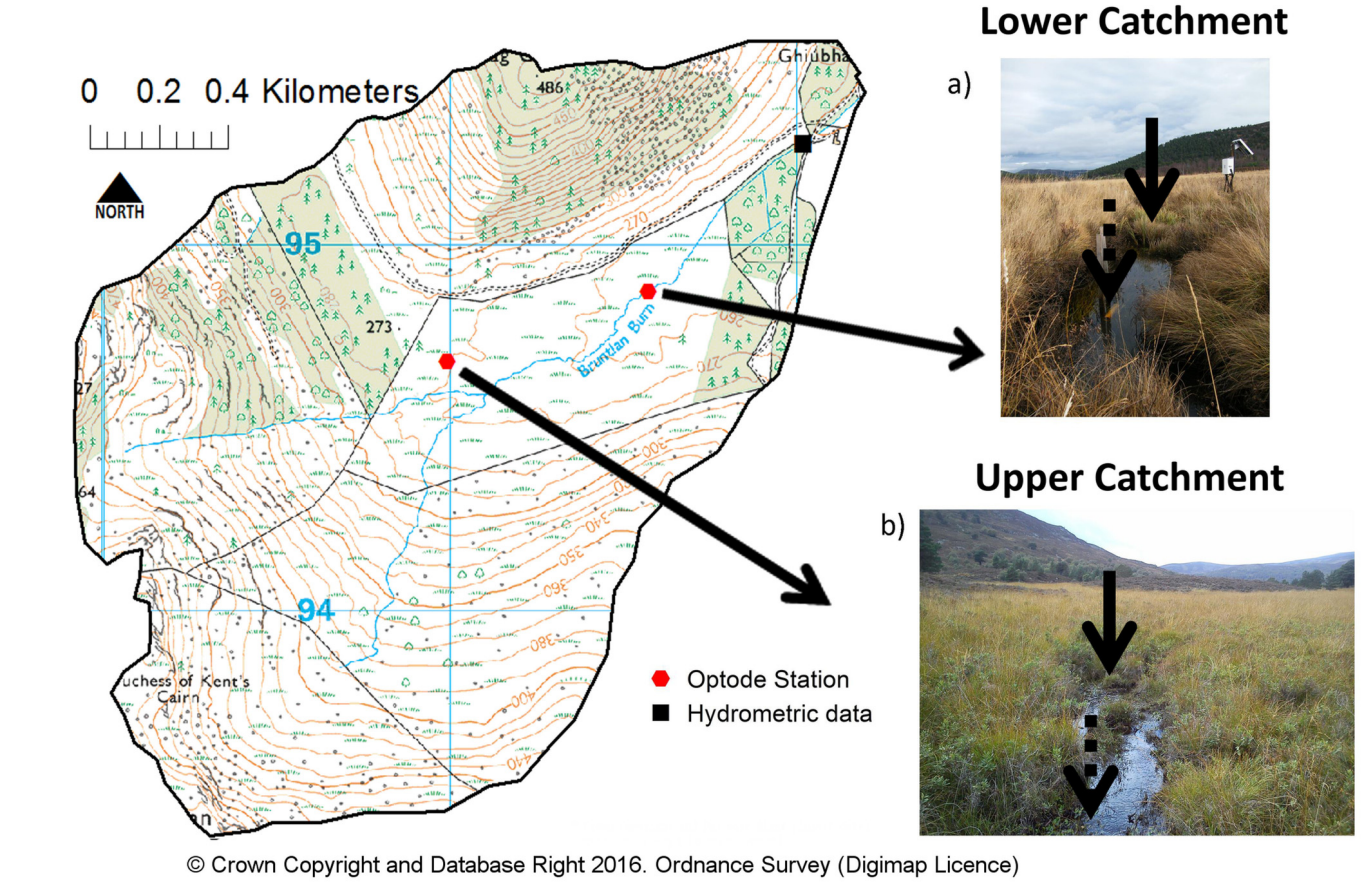
Topography of the study catchment and location of hydrometric data (black square) and optode dissolved oxygen measurement locations (red hexagon). a) photograph of lower catchment location b) photograph of upper catchment location. Arrows on the photographs show the approximate location of optodes. Dashed arrow = upstream. Solid arrow = downstream.

The area has been glaciated, and the stream occupies an over-widened, gently sloping valley (Fig 1), which limits the effects of topographic shading. As with many UK upland catchments, the Bruntland Burn is an open moorland stream with limited forest cover (Fig 1). The dominant vegetation in the riparian zone includes *Sphagnum spp*. mosses, dwarf shrubs (*Calluna vulgaris*, *Myrica gale*) and grasses (*Molina caerulea*), which become denser and taller in the lower catchment, with the higher vegetation clear in Fig 1a and 1b. Both locations had macrophyte growth in the stream channel. Although this was not measured quantitatively, the much greater biomass at the lower site was visually obvious. The dominant macrophytes at both sites were: bog pondweed (*Potamogeton polygonifolius)*, bulbous rush (*Juncus bulbosus*), marsh horsetail (*Equisetum palustre*), bottle sedge (*Carex rostrata*), and small bur-reed (*Sparganium minimum*). Only found in the lower catchment were: Common marsh-bedstraw (*Galium palustre*), and lesser spearwort (*Ranunculus flammula*).

The average annual precipitation is ~1000 mm with a low mean annual evapotranspiration of ~ 400 mm. Mean annual air temperatures are about 6°C, ranging between 12°C and 1°C in summer and winter respectively. Snow occurs, but usually comprises < 5% of the annual precipitation, though it was more prevalent during the study period. Precipitation is usually fairly evenly distributed, with limited seasonality, but with most falling in low intensity frontal events (around 50% of the precipitation falls in events of <10 mm). Most precipitation events instigate a stream flow response, as water enters the stream as saturation-excess overland flow from the saturated riparian areas, which are quasi-continuously connected to the channel network [36]. The stream channel has generally eroded through the peat and the stream bed is minerogenic and armoured by boulders and other coarse sediments in the glacial drift. Thus the channel generally meanders with high hydraulic roughness at both the micro- and macro-scales.

The upper catchment (UC) site is at the outflow of a large mire, and the channel has a pool-riffle morphology. The lower catchment (LC) site is on the main Bruntland Burn (Fig 1), and was again of pool-riffle morphology, though channel dimensions are different (Table 1). Locations were chosen in relatively homogenous reaches to limit the impacts of point source groundwater seeps with differential dissolved oxygen concentrations. Groundwater inflows account for around 30% of annual runoff from the Bruntland burn. Inflows are typically diffuse, can have relatively high DO concentrations and can rapidly mix within the hydraulically rough channel network [37]. Most runoff (~70%) is derived from surface flows during storm events from the fringing peats soils; these can have long surface flow paths re-equilibrating with atmospheric O_2_ before reaching the stream, thus minimising localised inputs of reduced water [38]. These hydrological factors are likely to have a minimal influence on the stream dissolved oxygen compared with re-aeration and in-stream metabolism.

## Data and Methods

### Monitoring

The monitoring period from 14^th^ July 2012 to 1^st^ November 2013 covered both growing and non-growing seasons. DO was monitored at the upper and lower catchment sites by two optical sensors (Aandera 3830 optodes) installed at each location (connected to a CR23X data logger and powered using a solar panel). They were situated at each location, around 5m apart (for replication), and were laboratory calibrated prior to installation. They were found to have a precision of 0.1°C and 1% DO. During the study period, both optodes at each location were placed in an aerated bucket to check that they maintained the same % saturation, which was found to be within 1–2%. Localities were chosen to enable a meaningful catchment scale comparison between the 1^st^ and 2^nd^ order streams, and were selected based on the similar channel morphology (common with the majority of the channel network) and similar topography (e.g. hillslope shading). All optodes measured in-stream DO percentage saturation and temperature at 15 minute intervals.

Discharge was measured at the catchment outlet (Odyssey data recording capacitance water level logger with 0.8mm resolution). This data was supplemented by meteorological data from an automatic weather station (AWS) 3km away, operated by Marine Scotland Science [39], from which air temp and shortwave radiation was used. All data is measured at 15 minute intervals. Air pressure at mean sea level was used from the Met Office weather station in Aboyne (Aboyne No. 2), which is located 20km from the catchment outlet.

All optode time series had data gaps, mainly 31^st^ May 2013–22^nd^ October 2013 at the upper catchment and 21^st^ December 2012–14^th^ February 2013 in the lower catchment, along with shorter periods in the order of a few days. These periods were identified during weekly field visits and through manual inspection of the data. All periods with lost or suspect data were removed from the time series. Reasons for data gaps include: the removal of a large period of data during winter 2012–2013 at the lower catchment location due to sustained snow cover over the solar panel resulting in power failure, optode icing during the winter of 2012–2013, dewatering at the upper catchment location from June 2013, and siltation, which became a problem after the snow thawed in spring 2013 (which lead to removal of the data from analysis). Due to the modelling approach, where data gaps existed, the full period was removed in 24 hour sections. As a result of the data gaps occurring during different time periods, both time-series are only broadly comparable, statistical analysis was conducted only on periods where data existed at both sites.

DO was measured as percentage saturation (DO_sat_) following Malcolm et al. [40], and thus it was necessary to convert it into concentration [mg l^-1^], and for this, a similar method to Demars et al [41] in Eqs 1–5.

$$DO_{o}=\frac{DO_{sat}-O_{sol}}{100}$$ (1)

For this it is necessary to calculate the oxygen solubility *O*_*sol*_ [mg O_2_ l^-1^](Eq 2) using altitudinally corrected pressure *P*_*alt*_ [Pa](Eq 3), the saturation water vapour pressure *V* [Pa](Eq 4) and the oxygen solubility under normal conditions (Eq 5) $O_{sol}^{atm}$ [mg O_2_ l^-1^]:

$$O_{sol}=\frac{O_{sol}^{atm}\left(P_{alt}-V\right)}{\left(101.325-V\right)}$$ (2)

$$P_{alt}=P_{msl}e^{-\left(\frac{mgE}{kT_{a}}\right)}$$ (3)

Eq 3 calculates the altitudinally corrected pressure *a* < *P* using: *P*_*msl*_ (the pressure at mean sea level in Pa); *m* (the molecular mass of dry air); *g* (the acceleration due to gravity) [m s^-2^]; elevation (*E*)[m]; *k* (the Boltzmann constant); *T*_*a*_ (air temperature in °C).

$$V=0.00005T_{a}^{3}+0.001T_{a}^{2}+0.0473T_{a}+0.6089$$ (4)

$$O_{sol}^{atm}=0.00008T_{s}^{3}+0.008T_{s}^{2}-0.404T_{s}^{}+14.609$$ (5)

Eq 5 additionally requires the stream temperature *T*_*s*_.

### Modelling approach

The study utilised an oxygen mass balance model (Eq 6) to calculate stream productivity, respiration and re-aeration by calibrating the measured diel oxygen curves [25].

$$\frac{\partial DO}{\partial t}=\frac{RC+P-R}{h}$$ (6)

Where *RC* is re-aeration [mg O_2_ l^-1^ h^-1^]; *P* is productivity [mg O_2_ l^-1^ h^-1^]; *R* is respiration [mg O_2_ l^-1^ h^-1^] and *h* is stream depth [m].

To calculate *h*, the mean stream depth [m] at each of the optodes, discharge was measured at each location by measuring channel dimensions and incremental velocities. Using the channel width (*W*)[m], the discharge at the outlet (*Q*) [m^3^ s^-1^] and velocity (*V*) [m s^-1^], *h* was derived from the hydraulic relationship between gauged discharge and the stream velocity at the outlet with that measured at each of the sites using gauged measurements (Eq 7):

$$h=\frac{Q}{VW}$$ (7)

A single station approach was adopted, (e.g. [8,19,42]). Though the single station method was envisaged from the outset, two optodes were installed at each site for replication. This also allowed comparison within each location.

The model was run for averaged hourly data for the two optodes at the upper catchment site (UC1 and UC2) and the two from the lower catchment site (T1 and T2). Hourly averages were chosen to remove noise from anomalous 15 minute measurements introduced by rapidly changing environmental conditions rather than slower reacting ecosystem processes, similar to [25,43,44]. The model uses air temperature, stream water temperature, incoming shortwave radiation and air pressure at mean sea level along with DO as the input variables.

#### Re-aeration

Re-aeration of the stream (oxygen exchange occurs across the water-atmosphere boundary), was calculated using the function given in Eq 8.

$$R=K_{a}T_{s}\left(D\right)$$ (8)

*RC* [mg O_2_ l^-1^ h^-1^] is the function of the re-aeration coefficient (*K*_*a*_) [h^-1^] (Eq 9), Temperature (*T*_*s*_) [°C] and the oxygen deficit (*D*)[mg l^-1^] which is given by Eq 10.

$$K_{a}T_{s}=K_{a}\theta^{T_{s}-20}$$ (9)

Where *θ* is set to 1.0241 to adjust the re-aeration coefficient for temperature [45]. To calculate the oxygen deficit *D* the observed oxygen (*DO*_*o*_) [mg l^-1^] is subtracted from the saturated oxygen (*DO*_*sat*_) [mg l^-1^] per hourly time step [46]. A positive *D* indicates an oxygen deficit and a negative *D*, super saturation of oxygen.

Due to the logistics of repeated gas evasion methods in remote sites, such as the one studied and controversial results using empirical equations [22], the re-aeration coefficient (*K*_*a*_) was treated as a calibrated model parameter similarly to Holtgrieve et al. [25]. Due to the importance of re-aeration, and our use of a relatively novel method, an indicative comparison with modelled re-aeration against that calculated using the night time regression method of Hornberger and Kelly [24] was carried out. This uses the night time decrease in dissolved oxygen concentrations and has been widely used to estimate re-aeration (e.g. [47]).

#### Stream respiration

The in-stream respiration (*R*)[mg O2 l^-1^ h^-1^] is estimated using a non-linear function which accounts for day time and night time respiration [48]:

$$R=\left(R_{20}+\beta I\right)\theta^{T_{s}-20}$$ (10)

Here, *β* [mg O_2_ W^-1^ h^-1^] relates respiration rates to the amount of light received and *R*_20_ [mg O_2_ l^-1^ h^-1^] the rate of respiration at 20°C. The *β* parameter can be set to 0 assuming no photo-respiration removing *βI*, and thus reduces the equation to a single parameter. *I* is the incoming radiation [W m^-2^].

#### Stream primary production

Photosynthetic production (*P*)[mg O_2_ l^-1^ h^-1^] was conceptualised following the methods of [49] allowing a switch between the linear parameter *P*_*1*_ [(mg O_2_ W^-1^ h^-1^)^-1^] and non-linear light saturation parameter *P*_2_ [(mg O_2_ W^-1^ h^-1^)^-1^] (Eq 11):

$$P=\frac{I}{P_{1}+P_{2}I}$$ (11)

The first oxygen concentration values measured (at 01:00:00) were assigned as initial values for each day’s model, and excluded from evaluation.

Hourly photosynthetic production, respiration and re-aeration were integrated to estimate daily gross photosynthetic production (GPP) [mg O_2_ l^-1^ d^-1^], daily ecosystem respiration (ER) [mg O_2_ l^-1^ d^-1^] and daily re-aeration (RC) [mg O_2_ l^-1^ d^-1^].

### Model calibration and selection

In total, five calibrated parameters were used to solve the oxygen mass balance: *K*_*a*_, *R*_20_, *β*, *P*_1_, *P*_2_. They were iteratively calibrated to individual 24 hour periods (411 days at the lower catchment site and 321 days at the upper catchment site), by running through the full DO time series using the input climate variables to drive the simulations. It was assumed that during each 24 hour period each parameter remained constant, but variable environmental conditions may have an effect on the ecosystem processes over longer time scales similar to findings by Mulholland et al. [13] and Reichert et al. [50]. The iterative model fitting produces time-variable parameter sets that might reflect changing ecosystem processes in response to variable environmental drivers (see section 4.2 below). The model was optimized using a differential evolution (DE) optimization algorithm according to [51], set to minimize the root mean squared error (RMSE)[mg l^-1^] as an objective function:

$$RMSE=\sqrt{\frac{\sum_{i=1}^{n}{\left(DO_{obs}-DO_{sim}\right)}^{2}}{n}}$$ (12)

Whilst not expected to produce a perfect solution, the DE algorithm provides a parameter set representative of the optimal parameter set, within the defined parameter space [26]. Model residuals were used as measures of likely parameter uncertainty, in the form of simulation bounds. A “rejectionist” framework was used, with stringent criteria used to evaluate simulations and only retain the best as “behavioural”. (i.e. daily simulations were assumed to be representative with the modelled processes fitting the diel DO curve). Models that do not fit the criteria are rejected as “non-behavioural”, as the diurnal oxygen dynamics deviated from a ‘steady state’ sinusoidal pattern due to processes such as rapid changes in stream flow, which were not predicted by the model. Simultaneously, parameter co-linearity was calculated based on Brun et al. [52], and implemented in the manner of Soetaert et al. [53]. This assesses the linear dependence of parameters, with co-linearities >20 being less identifiable. Identical selection criteria was used at both sites to avoid biasing. It was assumed that models not meeting these criteria are non-behavioural, with influencing factors which the model is unable to represent, being responsible for the diel oxygen curve. The stringent criteria to avoid co-linearity are particularly important to meaningfully single-out the ecosystem processes driving the oxygen balance.

Daily parameter sets were only accepted as behavioural when:

1. The simulated DO curves, respiration (ER), gross primary productivity (GPP) and re-aeration (RC) contained physically reasonable values (i.e. no negative values).
2. Model residuals fell inside the 90^th^ and 10^th^ percentiles of observed versus modelled daily average DO.
3. The Co-linearity index was <20.
4. Simulated mean daily DO deviates by less than the 10th percentile of all simulations from the observed mean daily DO value.
5. The parameter values for each daily model did not fall on the boundaries of the initial ranges (Initial parameter ranges are given in Table 2).

**Table 2:** Parameter comparisons between upper catchment site and lower catchment site. K_a_ = Reaeration coefficient, R_20_ = Respiration rate, β = Photorespiration parameter, P_1_ = Linear photosynthesis parameter, P_2_ = Light saturation parameter.

				Lower catchment		Upper catchment	
Parameter	Units	Initial Range		LC1	LC2	UC1	UC2
*K*_*a*_	h-1	-10, 10	Mean	0.372	0.345	0.211	0.201
			*10*^*th*^	*2*.*62E-02*	*2*.*65E-02*	*8*.*38E-03*	*1*.*21E-02*
			*90*^*th*^	*8*.*36E-01*	*7*.*98E-01*	*3*.*81E-01*	*3*.*82E-01*
*R*_*20*_	mg O_2_ l^-1^ h^-1^	0, 2	Mean	0.259	0.236	0.138	0.118
			*10*^*th*^	*7*.*22E-07*	*1*.*30E-06*	*1*.*22E-02*	*2*.*88E-03*
			*90*^*th*^	*5*.*74E-01*	*5*.*74E-01*	*2*.*34E-01*	*2*.*07E-01*
*β*	mg O_2_ w^-1^ h^-1^	0, 1	Mean	0.004	0.007	0.006	0.022
			10^th^	4.50E-05	2.32E-04	8.74E-11	2.72E-05
			90^th^	9.62E-03	8.08E-03	4.23E-03	5.54E-03
*P*_*1*_	(mg O_2_ w^-1^ h^-1^)^-1^	0, 5000	Mean	610.077	671.277	1164.077	1335.859
			*10*^*th*^	*3*.*95E-04*	*3*.*35E-04*	*4*.*20E+00*	*7*.*54E+00*
			*90*^*th*^	*1*.*54E+03*	*1*.*80E+03*	*2*.*64E+03*	*2*.*75E+03*
*P*_*2*_	(mg O_2_ w^-1^ h^-1^)^-1^	0, 50	Mean	8.216	8.628	8.940	12.683
			*10*^*th*^	*4*.*72E-03*	*9*.*52E-03*	*6*.*17E-03*	*2*.*02E-02*
			*90*^*th*^	*2*.*47E+01*	*3*.*32E+01*	*2*.*61E+01*	*3*.*77E+01*

Rejected models were viewed as being informative in terms of reasons for failure, and thus were assessed against the extremes of potential in-stream stressors such as flow, temperature and water quality.

The daily net ecosystem productivity (NEP), which is primary production minus respiration, was converted into units of carbon (C) (NEP_c_). This was done by converting oxygen units into units of carbon using the oxygen to carbon mole ratio of 0.375 (12 mgC/32mgO_2_) and assuming P/ER ratio of around 1 [54;55]. To assess the inter-site and intra-site differences and relationships with environmental drivers and metabolic processes, bivariate plots and Spearman’s rank coefficients for modelled gross primary productivity (GPP), respiration (ER), re-aeration (RC) and discharge (Q), air temperature (T), incoming radiation (I) were used.

## Results

### Hydroclimate and DO data

**Fig 2:**
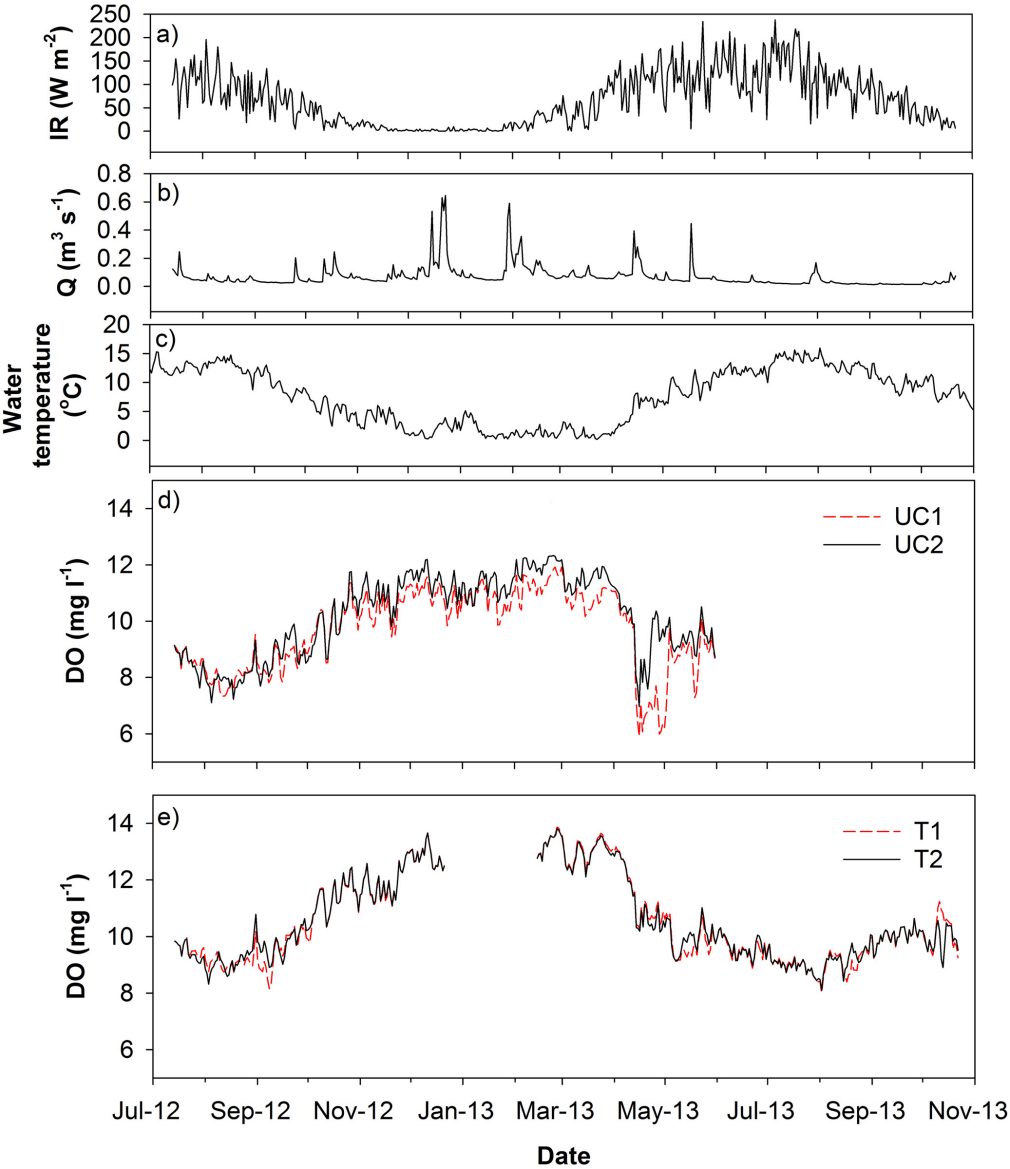
Mean Daily plots of: a) incoming shortwave radiation (IR), b) discharge (Q), c) water temperature at both sites, and dissolved oxygen for both the upper catchment (UC), panel d and the lower catchment (LC), panel e.

**Fig 3:**
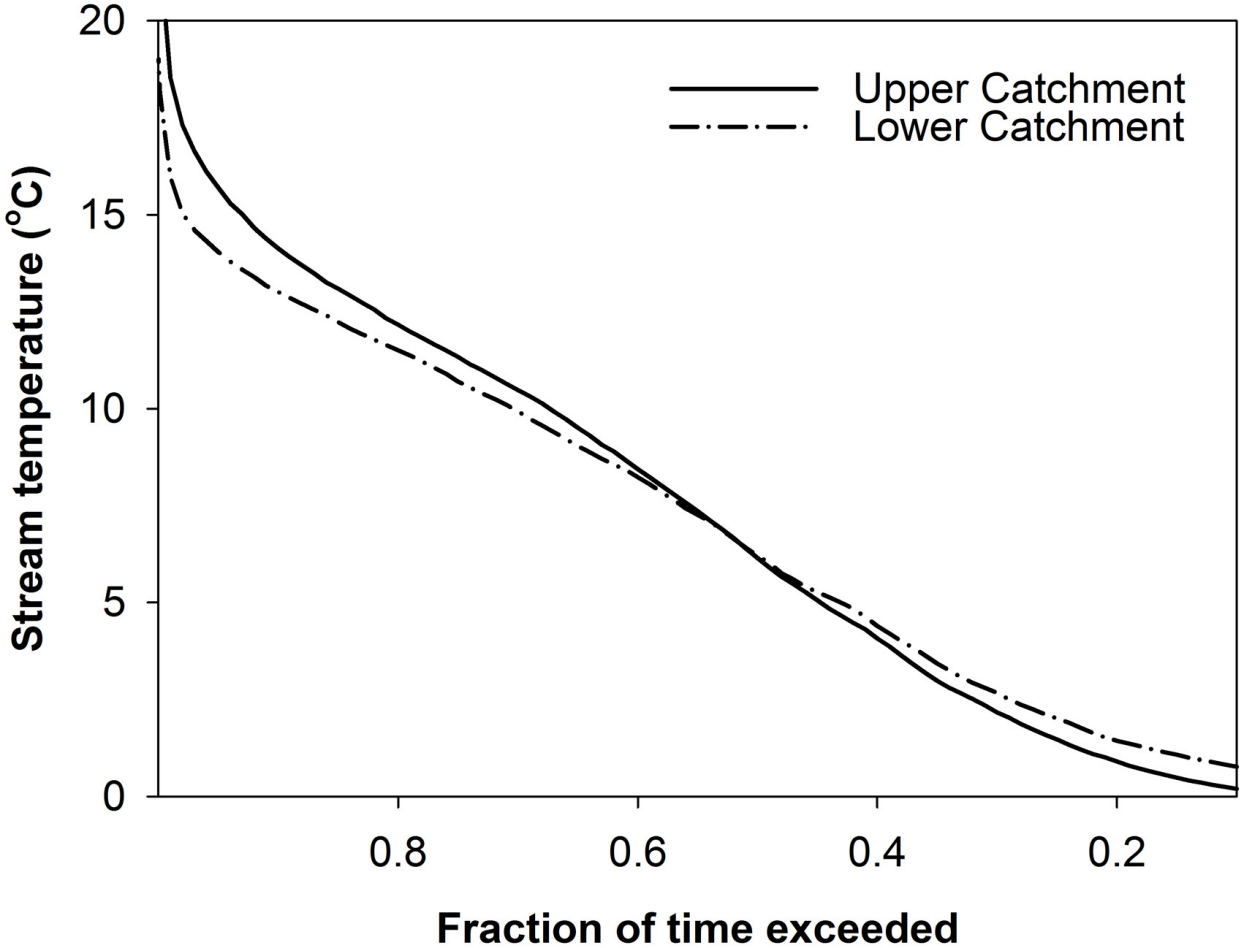
Stream temperature exceedance curve for the upper and lower catchment sites. This is the proportion of time at which a particular temperature is equalled or exceeded.

Hydrometeorological variation and dissolved oxygen dynamics over the study period (Fig 2) strongly reflect the seasonality of incoming shortwave radiation. This had the greatest variation and maximum values in the months of April to October (growing season) and was lowest with lowest variability during winter months (non-growing season). Precipitation was much lower in the summer of 2013 than 2012, with lower base flows in the stream reflecting this. Intermittent periods of snow cover (lasting from days to weeks) were experienced between December 2012 –April 2013 [56], typified by low flows observed during these periods, where in places the stream showed some surface icing. This was followed by melts which resulted in January and February high flows. High flows were subsequently experienced during spring to early-summer 2013, but it then became dry through into September until re-wetting in October. The dry period during summer 2013 led to dewatering of optodes at the upper catchment site and the mire became disconnected from the rest of the stream network. The lower catchment showed lower temperature variability (Table 1 and Fig 3), with attenuated thermal regimes of ~4.7°C standard deviation over the study period [57]. The upper catchment was generally more variable, with more time closer to the temperature extremes.

The lower catchment sites (LC1 and LC2) exhibited generally higher DO concentrations (average DO concentration 11.1 mg l^-1^ compared with 9.9 mg l^-1^ in the upper catchment during the comparable period of study) throughout the study period (Fig 2). Both the upper and lower catchment showed higher DO concentrations during the autumn and winter months (Oct-April) partly reflecting the water temperature differences (Fig 3). In the upper catchment, there was very similar variability between UC1 with a standard deviation of 1.40 mg l^-1^ and UC2 with a standard deviation of 1.42 mg l^-1^ (Table 1). UC1 generally exhibited lower DO concentrations following snowmelts in late winter. This may have reflected higher local rates of sedimentation of organic material in the vicinity of this sensor. In the lower catchment sensors at both LC1 and LC2 recorded similar DO concentrations. Although, the dissolved oxygen at the lower catchment site was typically higher, the amplitude of the diurnal variability at both sites was similar (>1 mg l^-1^) but was highest during the summer.

### Modelling results

**Fig 4:**
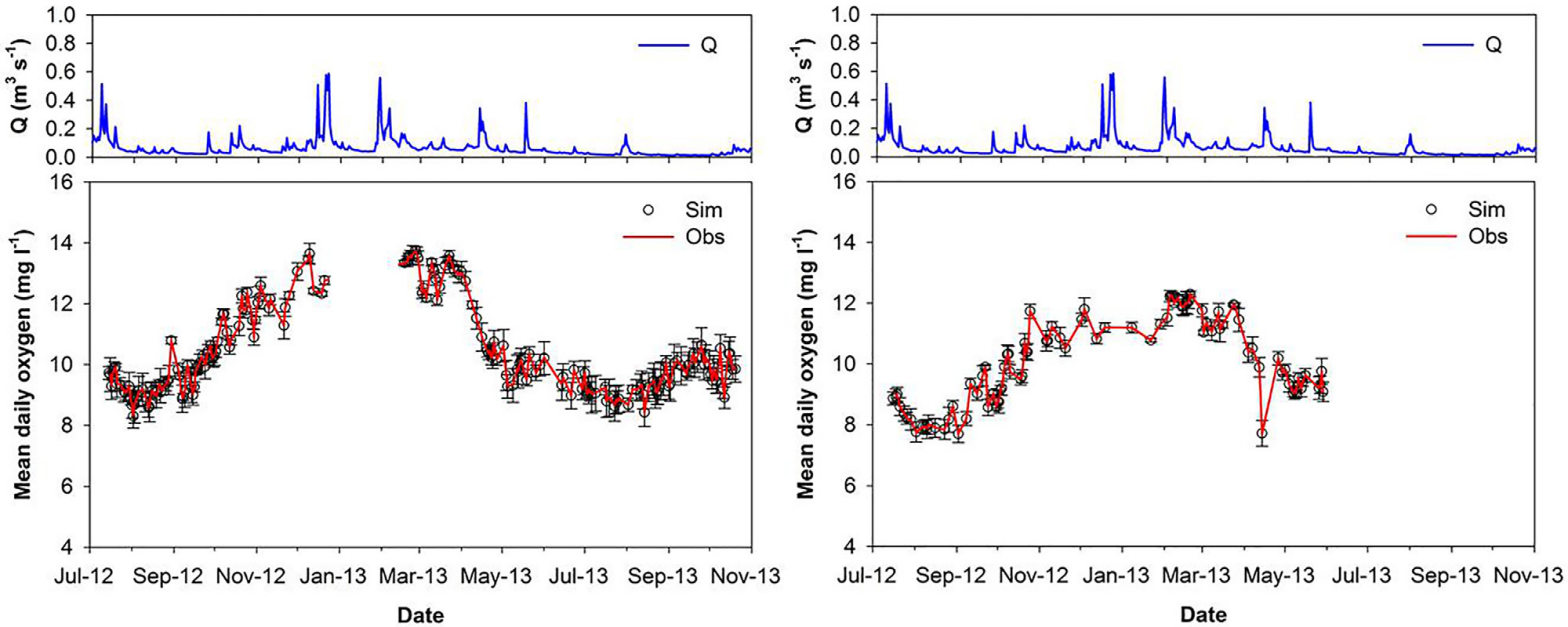
Discharge for the study period (top) with measured daily DO time-series compared to daily modelled DO with the daily RMSE as error bars. Lower catchment left, and upper catchment right.

**Fig 5:**
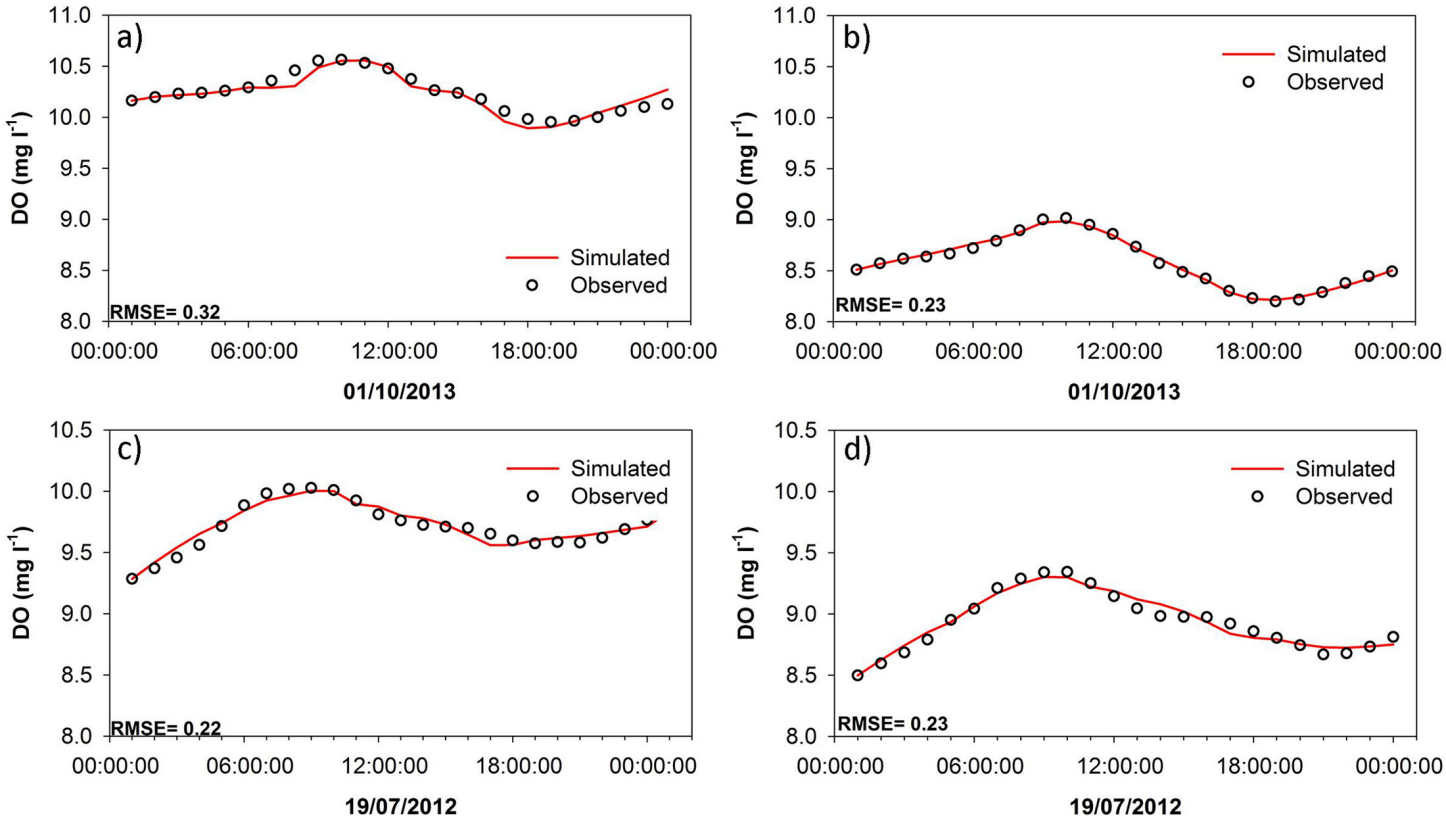
Simulated hourly DO versus the observed hourly DO at the upper catchment for a day during the non-growing season (01/10/2012) (a) and a day during the growing season (19/07/2012) (c). And hourly DO versus the observed hourly DO at the lower catchment for a day during the non-growing season (01/10/2012) (b) and a day during the growing season (19/07/2012) (d).

Given the rejectionist framework, the results for accepted model simulations in both the lower and upper catchment showed a good performance (Fig 4), with average RMSEs of 0.24 mg l^-1^ and 0.32 mg l^-1^ respectively. Inspection of the daily modelled versus observed DO showed that the accepted models fit the observed data well, with small RMSEs. These are evident in representative 24 hour periods for the non-growing (01/10/2012) and growing season (19/07/2012) for both the upper and lower catchment (Fig 5). It is clear that the accepted models simulated the diurnal variability well, during both summer and winter seasons, suggesting that the main metabolic processes were conceptualised in a plausible way by the algorithms used on these occasions.

However, the stringent model rejection criteria led us to discard simulations on 70% of the days (228 days) at the upper catchment site and 55% of the days (227 days) at the lower catchment site. Most rejected models were on days during the winter months which may be partially explained simply by limited diurnal variability in oxygen for which, the model is not able to differentiate the metabolic processes adequately. However, for most of the days when the models failed it did relate to more extreme environmental conditions. On almost half (111) of rejected days at the lower catchment location, flows were in the upper or lower quartile of the flow duration curve (i.e. above Q_25_ or below Q_75_); a further 20 days where models were rejected related to extreme temperature conditions in the upper and lower 5 percentiles (either >T_95_ 0r <T_5_). Finally, an additional 30 days of model rejection occurred when DOC concentrations (which were measured daily during the study year, see Dick et al., [35]) were above the 5^th^ percentile, which equates to 5.84 mg l^-1^. Overall, despite the higher rate of model rejection (which may in part relate to the greater availability of winter data), the upper catchment site showed generally better performance on days when models were accepted as behavioural, with a lower range in RMSE. Average RMSE for the UC was 0.24 mg l^-1^, and for the LC 0.32 mg l^-1^.

Despite the good performance of accepted models, the high number of rejected days and the data gaps, the inter-comparison of the time-series for both of the sites can only be used qualitatively (Fig 4). Nevertheless, analysis of the retained parameter values provides a basis for comparison of likely process differences between the sites (Table 2). A Wilcoxon rank sum test was carried out for significance on the differences between sites for days throughout the study period (n = 64) where calibrated models were accepted. Differences between the upper and lower catchment sites were significant (p <0.01) for the retained parameter sets for *K*_*a*_, *R*_*20*_ and *P*_*1*_. This indicates greater re-aeration at the lower catchment site, which is reflected in the higher values for the re-aeration coefficient, which would be consistent with the higher channel roughness and submerged macrophyte vegetation, which leads to greater turbulence [58]. The higher *R*_*20*_ in the lower catchment suggests that the location has a higher base level of respiration. These three parameters represent the simplest possible 3-parameter oxygen mass balance model described above and the other two parameters are clearly less well-identified.

**Fig 6:**
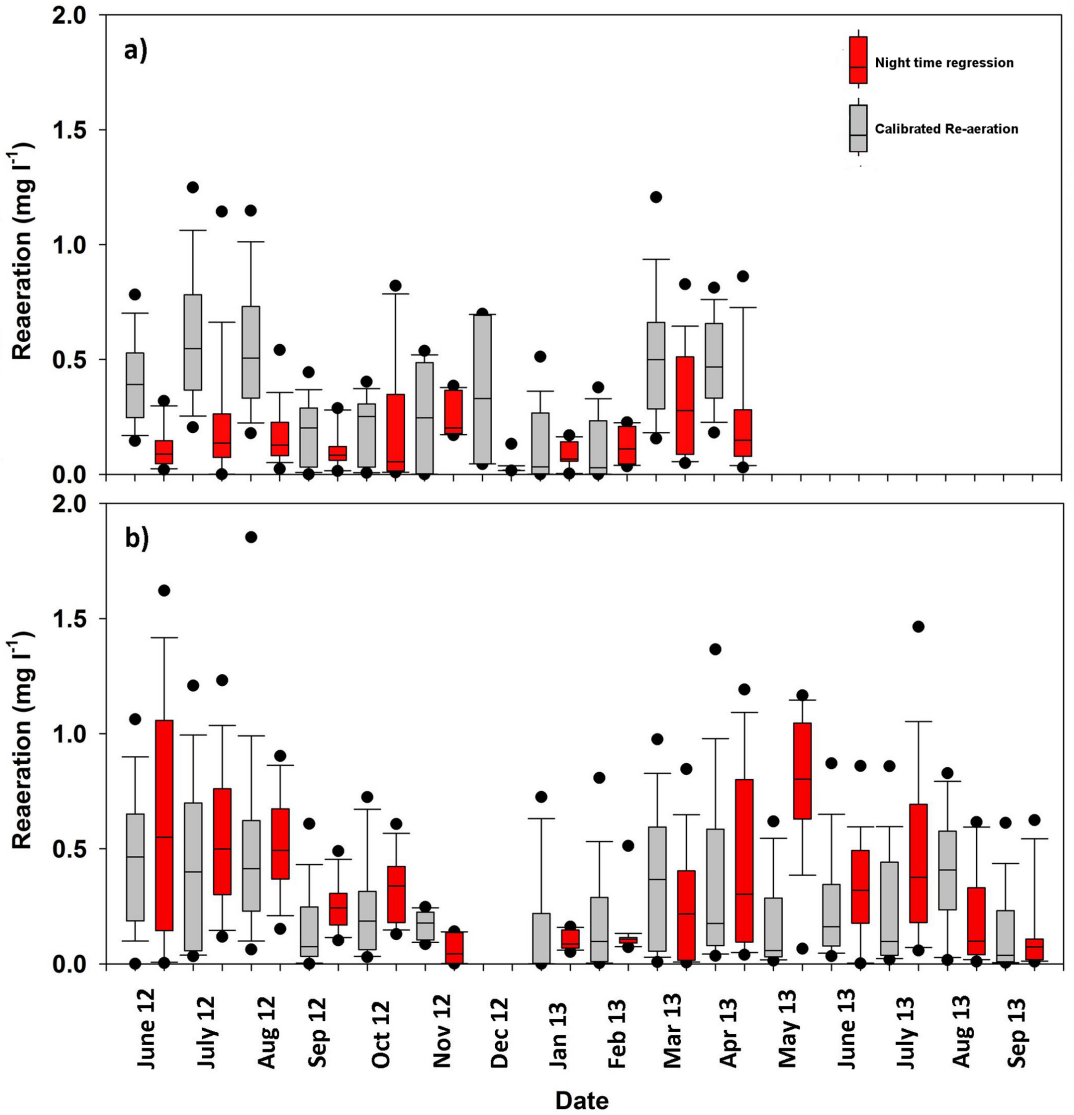
Monthly box plots comparing hourly re-aeration calculated using the calibrated re-aeration coefficient and the re-aeration coefficient calculated using the night time regression method [63] for the upper catchment (a) and lower catchment (b).

Due to the importance of re-aeration in metabolism modelling, the use of a calibrated re-aeration coefficient was expected to be a significant source of uncertainty [59]. Thus a comparison with the more commonly used (but not necessarily better) night-time regression method was carried out for LC2 and UC2 (Fig 6) taking into account that oxygen changes <1mg/l can limit the regression method. At the upper catchment location the re-aeration exhibited similar variability though with a lower monthly re-aeration coefficient, and at the lower catchment location the calibrated re-aeration coefficient was often higher than that using the methods of Hornberger and Kelly [24]. Ranges of the re-aeration coefficient (Table 2) were similar to the values presented by Demars et al. [60] for the deeper (> 0.1m water depth) Icelandic streams. Despite similar mean values and monthly dynamics, a comparison of the calibrated re-aeration with that calculated using the propane injection method [61], at a different (steeper) but nearby 1^st^ order upland stream (unpublished data by Benoit Demars, James Hutton Institute), also showed similar values (the difference between re-aeration was less than 20%) (Birkel, Personal comm.). As such the calibrated re-aeration parameter can be considered to be a useful first approximation [62].

**Fig 7:**
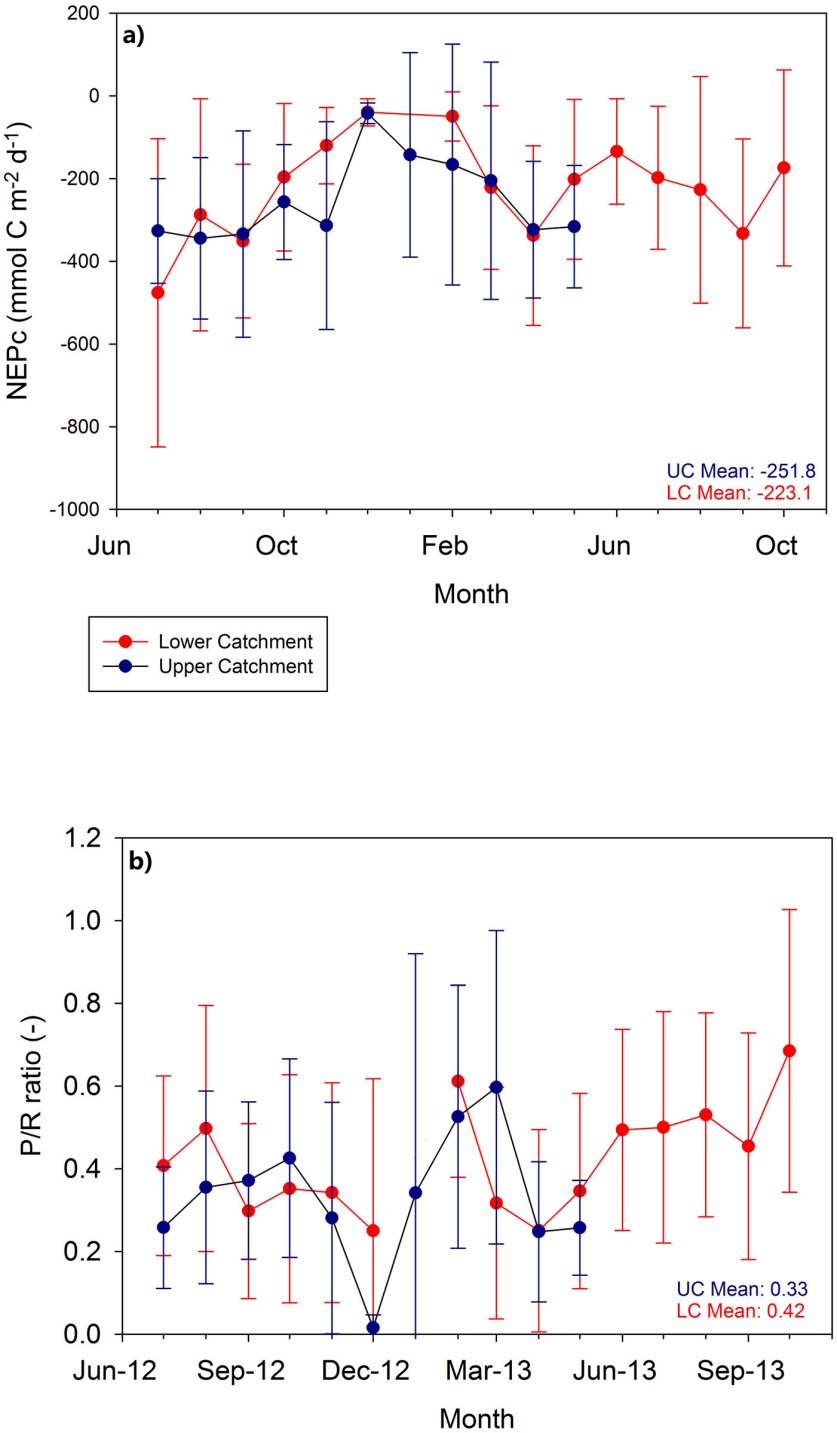
a) Mean monthly net ecosystem production converted into carbon units (NEP_c_) and b) the photosynthesis/respiration ratios for both the upper catchment and the lower catchment. Error bars are the standard deviation around the mean monthly values.

From the modelling, the components of in-stream metabolism could be estimated (Table 3). There is expected seasonality evident in the gross primary production (GPP) at both the upper and lower catchment sites, based on the daily mean GPP per month, with the lower catchment site exhibiting slightly higher average daily GPP per month during the growing season, though a Wilcoxon ranked sum test showed that these were not statistically different (p < 0.01) for the days where models were retained at both sites (Table 3). The mean daily GPP did show less variability at the upper catchment site throughout the period of data collection, though again this could be an artefact of a lower proportion of behavioural models. Similar seasonality was evident visually for respiration (ER) in the lower catchment, however, no consistent statistically significant differences between the sites were detected. Both the NEP_c_ and the P/R ratio also showed similar temporal patterns at each site (Fig 7). Average P/R ratios over the summer months in 2012 (June, July and August) were 0.40 and 0.33 at the lower and upper catchment sites respectively.

**Table 3:** Comparisons of the average and standard deviation of the daily gross primary productivity (GPP), ecosystem respiration (ER) and re-aeration (RC) for each month for both the lower and upper catchment. Both downstream loggers chosen at each location LC2 (lower catchment) and UC2 (upper catchment). The number of daily accepted models is also included at the right hand side of the table. Shaded areas represent periods where there is no data due to equipment malfunction.

	GPP (mg O_2_ l^-1^ d^-1^)				ER (mg O_2_ l^-1^ d^-1^)				RC (mg O_2_ l^-1^ d^-1^)				Accepted days	
	Lower Catchment		Upper Catchment		Lower Catchment		Upper Catchment		Lower Catchment		Upper Catchment			
	Mean	St.Dev	Mean	St Dev	Mean	Std Dev	Mean	Std Dev	Mean	Std Dev	Mean	Std Dev	LC	UC
Jul-12	6.28	3.16	4.06	3.04	-18.97	11.40	-13.01	4.82	12.62	9.98	8.94	3.90	12	7
Aug-12	8.21	7.24	6.76	5.15	-16.43	10.08	-15.41	5.84	8.22	7.60	8.74	5.27	21	9
Sep-12	3.76	2.59	4.40	2.52	-13.12	6.08	-13.62	7.66	9.43	4.99	9.03	6.99	21	10
Oct-12	2.20	1.26	4.57	1.39	-7.76	4.96	-11.29	3.61	5.62	5.01	6.70	3.95	17	14
Nov-12	1.41	0.96	3.04	1.95	-4.62	2.63	-13.28	8.61	3.31	2.31	10.66	7.85	8	5
Dec-12	0.26	0.30	0.02	0.03	-1.31	0.93	-1.13	0.67	1.05	0.88	1.08	0.86	6	4
Jan-13			0.18	0.18			-3.98	6.60			4.14	6.48		3
Feb-13	1.67	2.51	1.89	1.12	-3.15	3.98	-6.69	8.09	1.48	1.77	4.83	8.12	8	12
Mar-13	1.66	1.57	3.99	1.81	-7.95	5.94	-9.56	6.76	6.21	5.45	5.23	7.10	16	10
Apr-13	2.51	2.03	4.56	3.40	-11.97	7.27	-14.46	6.30	9.33	6.28	9.47	5.03	12	6
May-13	3.71	2.95	3.17	2.06	-9.39	7.37	-13.20	3.52	5.88	5.96	9.96	3.11	13	14
Jun-13	8.24	5.99			-12.81	8.74			4.77	4.22			7	
Jul-13	6.17	4.06			-11.58	5.34			5.41	4.60			14	
Aug-13	4.74	3.59			-10.96	9.35			6.32	7.44			16	
Sep-13	7.92	5.82			-17.00	7.49			9.11	6.28			14	
Oct-13	6.46	4.98			-11.10	9.38			4.66	6.23			14	

**Fig 8:**
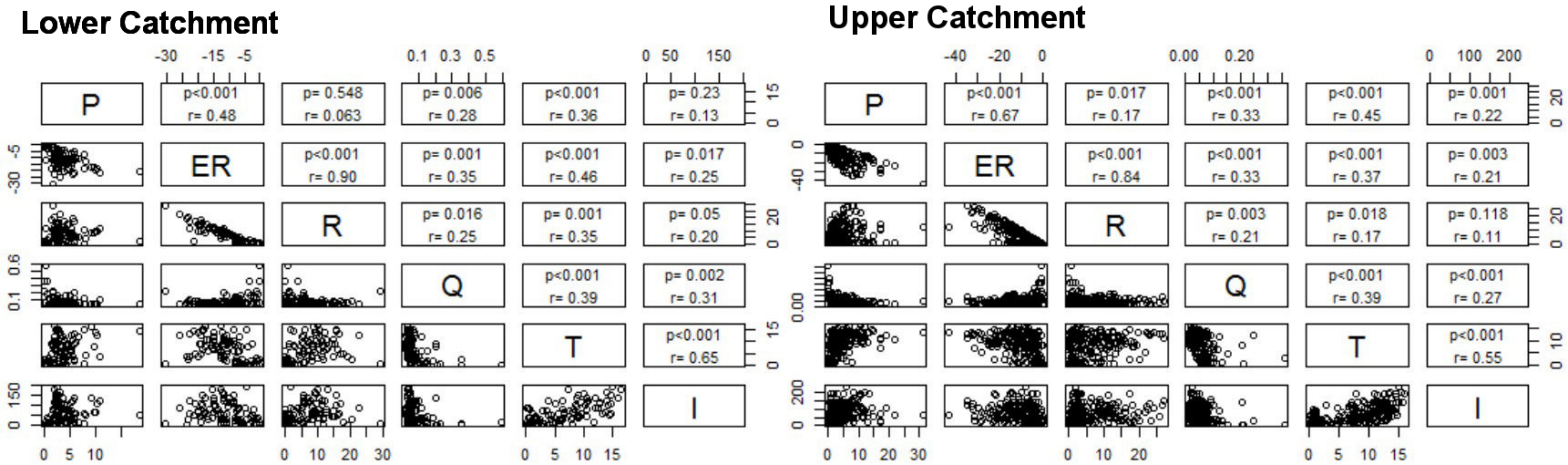
Bivariate plots of daily gross primary productivity (GPP), respiration (ER), re-aeration (RC), discharge (Q), water temperature (T) and incoming shortwave radiation (I) from accepted models for both the upper catchment (right) and the lower catchment (left). r = correlation coefficient, p = significance level.

Comparing and correlating the components of stream metabolism and environmental drivers for the days with accepted models (Fig 8) showed only weak relationships between re-aeration and discharge at either the upper (r = 0.25) or lower catchment (r = 0.21) indicating a limited effect of changing hydraulic conditions on reaeration probably because channel roughness creates turbulence at both high and lower flows. Significant (p = <0.001) correlations were evident for GPP and ER and between ER and RC at both sites, with greater productivity apparently fuelling greater respiration. Though this may also reflect the similar (and opposite) roles of R and RC in the mass balance equations, especially when the diurnal variation in DO is small. The lower catchment showed significant correlations (at both optodes) between productivity and water temperature (r = 0.45), but at the upper catchment site, correlations were lower (r = 0.36). Correlations between ER and RC and discharge, water temperature, and incoming radiation were generally higher for the upper catchment site, though this could be biased by missing data for summer 2013 (Table 3). Surprisingly, given the channel geometry (channel width), the relationship between incoming radiation and primary production was higher at the lower catchment site (r = 0.22 versus 0.13 at the UC site). The factors included (Q, T and I) were also model inputs, and as such, relationships with the modelled outcomes are not unexpected, but the tentative difference in the strength of the correlation is of interest. Again, care is also needed, due to the significant difference in the number of accepted daily models and timing of data gaps.

## Discussion

### How well does the model capture metabolism in moorland peat streams?

The number of accepted models for both locations was relatively low (45% acceptance rate in the lower catchment and 30% in the upper catchment), however the results are still valuable, as this is a novel use of a oxygen mass balance model with a calibrated re-aeration flux in 1^st^ order peat streams and 2^nd^ order upland catchments [26]. Model failure results from deviation of the DO from a sinusoidal pattern due to environmental factors such as temperature and flow extremes. This in turn leads to the daily model failing to meet the model acceptance criteria. This high rejection rate, is a corollary of the aims of the study in applying a simple behavioural model to DO data, in order to estimate stream metabolism, and investigate its dynamics. Upland streams are known to be very dynamic systems, with short response times to changing conditions. There are many factors which may change the oxygen balance and metabolic processes in streams occurring. Such factors include variation in dissolved organic matter inputs [64], changes in stream temperatures [65,41], scour during high flow periods [19,66], and the growth and decay of macrophytes [67]. As shown, almost all of the days where models were rejected had flows that were in the upper or lower quartile of the distribution, temperatures that were in the upper or lower 5 percentile or DOC concentrations in the upper 5 percentile, due to light limitation by the DOC (coloured) water. Thus the retained mass balance models appear to be most appropriate where hydrological and hydroclimatic conditions are closer to average for which they are only strictly valid.

The results indicated that using the simple mass balance model with a calibrated re-aeration produced reasonable performance and relatively high parameter identifiability for both sites. This is shown in the constrained calibrated ranges for most parameters compared with the initial ranges in Table 2. This indicated that the model plausibly captured the processes that occur in the stream and provided a reasonable estimation of the balance between respiration, production of oxygen via photosynthesis, and atmospheric re-aeration. There was a slightly poorer fit of daily models to the lower catchment location diel oxygen curves, though both showed very low residuals (<0.1 mg l^-1^ difference at upper catchment site and <0.2 mg l^-1^ difference in the lower catchment).

The model was previously used and developed for the larger Girnock catchment [26], which has a more flashy response to precipitation and greater turbulence. The lower amounts of re-aeration in the Bruntland Burn (lower re-aeration coefficients than in the Girnock catchment with 10^th^ and 90^th^ percentiles of 0.01 and 0.9 respectively) are consistent with a lower gradient and differences in channel characteristics, which is narrower and deeper and with greater bed armouring. In the Girnock, there is increased turbulence and as such, greater re-aeration [68].

General similarities between the calibrated and calculated re-aeration suggest that, within the Bruntland Burn, the use of a calibrated re-aeration parameter is reasonable and has some advantages over manual methods of calculating re-aeration, which (like night-time regression) can also be uncertain or are often not practical as they are physically difficult and expensive to repeat across a range of conditions at remote sites [69]. The differences between the calculated and calibrated methods could be due to the underlying assumption in the night time regression method [63] that respiration remains constant during the night, when changes in water temperature might make this inappropriate, or the DO change is <1 mg l^-1^ causing uncertainty in the method. However, the introduction of re-aeration as a calibrated parameter, introduces uncertainty within the model as GPP, respiration and re-aeration can covary and produce similar results. This allows different rates of metabolism and re-aeration, which produce the same diel signal of DO [69]. Despite this model equifinality, the stringent rejectionist framework selected the most plausible models in line with the simplifying model assumptions. Though re-aeration is the main source of uncertainty in modelling stream metabolism, this approach enables qualitative interpretations into system dynamics to be made. A further limitation of the use of a simple behavioural model is the inability to account for significant groundwater fluxes. To mitigate this, the sites were chosen in homogeneous reaches, with no point source groundwater influxes. Spatially distributed temperature monitoring in the same location did not indicate the presence of large localised points of groundwater input [70].

### What does the stream metabolism tell us about the system?

There has been relatively little work on metabolic processes in 1^st^ order and 2^nd^ order, peat bounded streams [10]. Most 1^st^ and 2^nd^ stream order stream metabolism studies are located in very different climatic zones and landscapes, for example in arctic climates [60,64] and forested landscapes (c.f. [5,71]). Very few, if any, have focused on moorland streams draining mires, where organic soils dominate [72]. Most of the work regarding metabolic processes in these 1^st^ and 2^nd^ order peatland streams focuses on greenhouse gas evasion from such streams e.g. [73]. The lotic metabolic processes are important as well because they are usually the controlling factor in CO_2_ production [74,75] and are very important in the cycling and utilisation of organic matter within streams [76,77].

The P/R ratios define the metabolic balance of streams [78]. The estimated P/R ratios are consistently below one and show that the stream was heterotrophic at both sites, throughout the year. This is in keeping with most low productivity streams being heterotrophic globally [79]. There was greater heterotrophy in the summer, which is expected, given the increased instream decomposition and is in keeping with the findings of Schade et al. [15] and Birkel et al. [26] for headwater streams. Although direct comparison was difficult, there is a suggestion that the upper catchment more often has lower P/R ratios than the lower catchment and was more heterotrophic. This would be consistent with the greater productivity at the lower catchment site and as such, during the growing season primary productivity is greater as is the increased night time respiration parameter. NEP is based on the difference between the production and respiration estimates [13] and shows that both sites are sinks for organic matter and sources of CO_2_. This is in keeping with most of the world’s streams and rivers [80]. The slightly lower NEP_c_ in the upper catchment appears to hint at a greater source of CO_2_ than the lower catchment as a result of greater utilisation of available organic matter in mire runoff [81].

The study site exhibited mean NEPc of -251.8 mmol C m^-2^ d^-1^ in the upper catchment, and -223.1 mmol C m^-2^ d^-1^ in the lower catchment (Fig 7). In comparison, Glen Girnock had an average NEPc of -122 mmol C m^-2^ d^-1^ [26], this suggests increasing heterotrophy with stream order.

Birkel et al. [26], found that trees, as riparian vegetation, impacted the gross primary productivity (GPP) in Glen Girnock (of which the Bruntland Burn is a subcatchment) and created a greater seasonal signature. This is a finding supported by work looking at forested catchments, which found increased heterotrophy in forested reaches as a consequence of shade, limiting photosynthesis [82–84];. Lack of shrub cover may be responsible for the greater relationship between incoming radiation and productivity at the upper catchment site. A link between plant biomass and gross primary productivity was also found, and as biomass increases with stream order, this is consistent with the findings of this study and showed that primary productivity should be highest at the lower catchment with greater biomass [85] (higher stream order site). Although this was not quantitatively assessed, it is visually evident that the lower catchment contained greater macrophyte growth biomass. The study site exhibited mean NEPc of -251.8 mmol C m^-2^ d^-1^ in the upper catchment, and -223.1 mmol C m^-2^ d^-1^ in the lower catchment (Fig 7). In comparison, Glen Girnock had an average NEPc of -122 mmol C m^-2^ d^-1^ [26], this suggests increasing heterotrophy with stream order.

The wider implications of this work are that it provides insights into metabolism in peat-bound headwater streams, which are typically common throughout the uplands of the UK and in peat dominated landscapes in the upper northern latitudes. These streams have specific characteristics (linked to the hydrology and biology of the peatland), which have irregular geomorphology and velocity profiles [2]. Such streams are also likely to change in the coming decades. Climate change projections [86], even under low emission scenarios, predict a >1°C rise in temperatures, a 3% Increase in winter precipitation (and subsequent increase in runoff during the winter) and a 5% decrease in summer precipitation (and subsequent decrease in runoff). It is therefore increasingly important to monitor these locations as their open setting and biology may make them more sensitive to change [87], and knowledge of small scale controls will be important, in order to understand the implications that climate change will have [88].

## Conclusion

A mass balance model was used to simulate the spatially variable daily dynamics of stream metabolism, within first and second order peatland headwater streams. The main findings of this study were:

- 1^st^ and 2^nd^ order peat bounded streams are heterotrophic in keeping with larger systems, and as such, are sources of C to the environment.
- Mass balance models using dissolved O_2_ data in a rejectionist framework show that models perform best under steady state conditions and are unable to capture the perturbations caused by high and low flows and temperatures and high DOC concentrations.
- Calibrated model parameters show process differences between sites with the 2^nd^ order stream having higher productivity and respiration but losing less carbon x to the environment.
- Utilizing the re-aeration coefficient as a calibrated parameter provides a potentially effective tool for interpreting the dynamics of stream metabolism at small scales.

Future work is needed to identify the important controls of stream metabolism at the short time scales. This is due to the low correlations which were found between productivity and water temperature in this study, and may point to the smaller scale local processes (e.g. shading and vegetation) as dominant factors controlling metabolic processes. Use of longer periods of measurement and/or more spatial coverage in the sensor networks would enable an increase in our understanding of the factors which influence lotic metabolism in moorland streams.

## Supporting Information

S1 Dataset Supplementary data from the upper catchment location. (TXT)

S2 Dataset Supplementary data from the lower catchment location. (TXT)

S1 File Dissolved oxygen model code. (TXT)

## Abbreviations

DO: Dissolved oxygen [mg l^-1^]
P: Community production [mg O_2_ l^-1^ h^-1^]
GPP: Daily gross primary production [mg O_2_ l^-1^ d^-1^]
ER: Daily ecosystem respiration [mg O_2_ l^-1^ d^-1^]
R: Respiration [mg O_2_ l^-1^ h^-1^]
RC: Reaeration [mg O_2_ l^-1^ d^-1^
*P*_*alt*_: Pressure at altitude [Pa]
*P*_*msl*_: Pressure at sea level [Pa]
*m*: Molecular mass of dry air
*g*: Acceleration due to gravity [m s^-2^]
*V*: Vapour pressure [Pa]
$O_{sol}^{atm}$: Oxygen solubility under normal conditions [mg O_2_ l^-1^]
*O*_*sol*_: Oxygen solubility [mg O_2_ l^-1^]
t: Time
h: Stream depth [m]
Q: Discharge [m^3^ s^-1^]
V: Stream velocity [m s^-1^]
W: Stream width [m]
*K*_*a*_: Re-aeration coefficient [h^-1^]
*T*_*a*_: Air temperature [°C]
*T*_*s*_: Stream temperature [°C]
*I*: Incoming radiation [W m^-2^]
D: Oxygen deficit [mg l^-1^]
*DO*_*sat*_: Saturated DO [mg l^-1^]
*DO*_*o*_: Observed DO [mg l^-1^]
*R*_20_: Respiration rate parameter [mg O_2_ l^-1^ h^-1^]
*β*: Photorespiration rate parameter [mg O_2_ l^-1^ h^-1^]
*P*_1_: Linear photosynthesis parameter [(mg O_2_ l^-1^ t^-1^)^-1^]
*P*_2_: Light saturation parameter [(mg O_2_ l^-1^ t^-1^)^-1^]
RMSE: Root mean square error [mg l^-1^]
*DO*_*sim*_: Simulated DO [mg l^-1^]
*n*: Number of 24 hour periods
